# Supplementary material for: An open-source framework for large-scale, flexible evaluation of biomedical text mining systems
Source: J Biomed Discov Collab. 2008 Jan 29;3:1. doi: 10.1186/1747-5333-3-1 (PMC2276192; doi:10.1186/1747-5333-3-1)
Supplement: Additional file 1 — Evaluation platform source code. The additional file contains the software evaluation framework discussed in this paper. The most current release can be downloaded from SourceForge.net [1]. See the accompanying README file for instructions on installation and use. Also included in the distribution is a collection of UIMA wrappers for some commonly used BioNLP tools and annotated corpora. [file 1747-5333-3-1-S1.zip › bionlp_uima_repository-1.0.1/docs/api/edu/uchsc/ccp/util/nlp/stats/PRFResult.html]

PRFResult (Center for Computational Pharmacology Util/UIMA API)


|  |  |  |
| --- | --- | --- |
| **Overview** \| **Package** \| **Class** \| **Tree** \| **Deprecated** \| **Index** \| **Help** | |  |
| **PREV CLASS**   NEXT CLASS | **FRAMES**    **NO FRAMES**     **All Classes** |  |
| SUMMARY: NESTED \| FIELD \| CONSTR \| METHOD | DETAIL: FIELD \| CONSTR \| METHOD |  |


---


## edu.uchsc.ccp.util.nlp.stats Class PRFResult

```
java.lang.Object
  edu.uchsc.ccp.util.nlp.stats.PRFResult
```

---

``` public class PRFResult extends Object ```

This class is a data structure for cataloging the results of an NLP experiment. Not only are TP, FP, and FN counts logged, but the annotations that
were responsible for each TP, FP, and FN count are also recorded.

**Author:**
:   William A Baumgartner, Jr.

---

**Constructor Summary**

`PRFResult(int tp,
int fp,
int fn)`
  
          Initialize a new PRFResult by inputting counts for true positives, false positives, and false negatives.

`PRFResult(int tp,
int fp,
int fn,
String title)`
  
          Initialize a new PRFResult by inputting counts for true positives, false positives, and false negatives.


| **Method Summary** | |
| --- | --- |
| `void` | `add(PRFResult prf)`             Update this `PRFResult` object by adding the TP, FP, and FN counts from another `PRFResult` object. |
| `void` | `addFNAnnotation(TextAnnotation fnAnnotation)`             Add a false negative `TextAnnotation` |
| `void` | `addFPAnnotation(TextAnnotation fpAnnotation)`             Add a false positive `TextAnnotation` |
| `void` | `addTPAnnotation(TextAnnotation tpAnnotation)`             Add a true positive `TextAnnotation` |
| `int` | `getFalseNegativeCount()` |
| `int` | `getFalsePositiveCount()` |
| `double` | `getFmeasure()` |
| `List<TextAnnotation>` | `getFNAnnotations()`             Retrieve the annotations responsible for the false negative counts |
| `List<TextAnnotation>` | `getFPAnnotations()`             Retrieve the annotations responsible for the false positive counts |
| `double` | `getPrecision()` |
| `double` | `getRecall()` |
| `String` | `getTitle()` |
| `List<TextAnnotation>` | `getTPAnnotations()`             Retrieve the annotations responsible for the true positive counts |
| `int` | `getTruePositiveCount()` |
| `void` | `printAnnotations(PrintStream ps)`             Output to the given `PrintStream` the annotations responsible for the TP, FP, and FN statistics. |
| `void` | `printStats(PrintStream ps)`             Output to the given `PrintStream` the statistics recorded by this PRFResult |
| `void` | `setFalseNegativeCount(int falseNegativeCount)` |
| `void` | `setFalsePositiveCount(int falsePositiveCount)` |
| `void` | `setFNAnnotations(List<TextAnnotation> fnAnnotations)`             Set the false negative `TextAnnotation` list |
| `void` | `setFPAnnotations(List<TextAnnotation> fpAnnotations)`             Set the false positive `TextAnnotation` list |
| `void` | `setTitle(String title)` |
| `void` | `setTPAnnotations(List<TextAnnotation> tpAnnotations)`             Set the true positive `TextAnnotation` list |
| `void` | `setTruePositiveCount(int truePositiveCount)` |

**Methods inherited from class java.lang.Object**

`clone, equals, finalize, getClass, hashCode, notify, notifyAll, toString, wait, wait, wait`

**Constructor Detail**

### PRFResult

```
public PRFResult(int tp,
                 int fp,
                 int fn)
```

:   Initialize a new PRFResult by inputting counts for true positives, false positives, and false negatives.

    **Parameters:**: `tp` - a count of true positives: `fp` - a count of false positives: `fn` - a count of false negatives

---


### PRFResult

```
public PRFResult(int tp,
                 int fp,
                 int fn,
                 String title)
```

:   Initialize a new PRFResult by inputting counts for true positives, false positives, and false negatives.

    **Parameters:**: `tp` - a count of true positives: `fp` - a count of false positives: `fn` - a count of false negatives: `title` - a textual description of the NLP experiment under study


**Method Detail**

### getFmeasure

```
public double getFmeasure()
```

---


### getPrecision

```
public double getPrecision()
```

---


### getRecall

```
public double getRecall()
```

---


### getFalseNegativeCount

```
public int getFalseNegativeCount()
```

---


### setFalseNegativeCount

```
public void setFalseNegativeCount(int falseNegativeCount)
```

---


### getFalsePositiveCount

```
public int getFalsePositiveCount()
```

---


### setFalsePositiveCount

```
public void setFalsePositiveCount(int falsePositiveCount)
```

---


### getTruePositiveCount

```
public int getTruePositiveCount()
```

---


### setTruePositiveCount

```
public void setTruePositiveCount(int truePositiveCount)
```

---


### printStats

```
public void printStats(PrintStream ps)
```

:   Output to the given `PrintStream` the statistics recorded by this PRFResult

    :   **Parameters:**: `ps` - the `PrintStream` to use

---


### printAnnotations

```
public void printAnnotations(PrintStream ps)
```

:   Output to the given `PrintStream` the annotations responsible for the TP, FP, and FN statistics.

    :   **Parameters:**: `ps` - the `PrintStream` to use

---


### add

```
public void add(PRFResult prf)
```

:   Update this `PRFResult` object by adding the TP, FP, and FN counts from another `PRFResult` object. Note, at this
    time, the annotations do not get passed along, only the counts. This is for space considerations when evaluating large document collections.

    :   **Parameters:**: `prf` -

---


### getTitle

```
public String getTitle()
```

---


### setTitle

```
public void setTitle(String title)
```

---


### getFNAnnotations

```
public List<TextAnnotation> getFNAnnotations()
```

:   Retrieve the annotations responsible for the false negative counts

    :   **Returns:**: a list of `TextAnnotation` objects associated with the false negative counts

---


### addFNAnnotation

```
public void addFNAnnotation(TextAnnotation fnAnnotation)
```

:   Add a false negative `TextAnnotation`

    :   **Parameters:**: `fnAnnotation` -

---


### setFNAnnotations

```
public void setFNAnnotations(List<TextAnnotation> fnAnnotations)
```

:   Set the false negative `TextAnnotation` list

    :   **Parameters:**: `fnAnnotations` -

---


### getFPAnnotations

```
public List<TextAnnotation> getFPAnnotations()
```

:   Retrieve the annotations responsible for the false positive counts

    :   **Returns:**: a list of `TextAnnotation` objects associated with the false positive counts

---


### addFPAnnotation

```
public void addFPAnnotation(TextAnnotation fpAnnotation)
```

:   Add a false positive `TextAnnotation`

    :   **Parameters:**: `fpAnnotation` -

---


### setFPAnnotations

```
public void setFPAnnotations(List<TextAnnotation> fpAnnotations)
```

:   Set the false positive `TextAnnotation` list

    :   **Parameters:**: `fpAnnotations` -

---


### getTPAnnotations

```
public List<TextAnnotation> getTPAnnotations()
```

:   Retrieve the annotations responsible for the true positive counts

    :   **Returns:**: a list of `TextAnnotation` objects associated with the true positive counts

---


### addTPAnnotation

```
public void addTPAnnotation(TextAnnotation tpAnnotation)
```

:   Add a true positive `TextAnnotation`

    :   **Parameters:**: `tpAnnotation` -

---


### setTPAnnotations

```
public void setTPAnnotations(List<TextAnnotation> tpAnnotations)
```

:   Set the true positive `TextAnnotation` list

    :   **Parameters:**: `tpAnnotations` -


---


|  |  |  |
| --- | --- | --- |
| **Overview** \| **Package** \| **Class** \| **Tree** \| **Deprecated** \| **Index** \| **Help** | |  |
| **PREV CLASS**   NEXT CLASS | **FRAMES**    **NO FRAMES**     **All Classes** |  |
| SUMMARY: NESTED \| FIELD \| CONSTR \| METHOD | DETAIL: FIELD \| CONSTR \| METHOD |  |


---
